# Preventing bias from selective non-response in population-based survey studies: findings from a Monte Carlo simulation study

**DOI:** 10.1186/s12874-019-0757-1

**Published:** 2019-06-13

**Authors:** Kristin Gustavson, Espen Røysamb, Ingrid Borren

**Affiliations:** 1 Department of Mental Disorders, Norwegian Institute of Public Health, Oslo, Norway; 2 PROMENTA Research Center, Department of Psychology, University of Oslo, Oslo, Norway; 3 Department of Child Development, Norwegian Institute of Public Health, Oslo, Norway

**Keywords:** Selective non-response, Preventing bias, Monte Carlo simulations

## Abstract

**Background:**

Health researchers often use survey studies to examine associations between risk factors at one time point and health outcomes later in life. Previous studies have shown that missing not at random (MNAR) may produce biased estimates in such studies. Medical researchers typically do not employ statistical methods for treating MNAR. Hence, there is a need to increase knowledge about how to prevent occurrence of such bias in the first place.

**Methods:**

Monte Carlo simulations were used to examine the degree to which selective non-response leads to biased estimates of associations between risk factors and health outcomes when persons with the highest levels of health problems are under-represented or totally missing from the sample. This was examined under different response rates and different degrees of dependency between non-response and study variables.

**Results:**

Response rate per se had little effect on bias. When extreme values on the health outcome were completely missing, rather than under-represented, results were heavily biased even at a 70% response rate. In most situations, 50–100% of this bias could be prevented by including some persons with extreme scores on the health outcome in the sample, even when these persons were under-represented. When some extreme scores were present, estimates of associations were unbiased in several situations, only mildly biased in other situations, and became biased only when non-response was related to both risk factor and health outcome to substantial degrees.

**Conclusions:**

The potential for preventing bias by including some extreme scorers in the sample is high (50–100% in many scenarios). Estimates may then be relatively unbiased in many situations, also at low response rates. Hence, researchers should prioritize to spend their resources on recruiting and retaining at least some individuals with extreme levels of health problems, rather than to obtain very high response rates from people who typically respond to survey studies. This may contribute to preventing bias due to selective non-response in longitudinal studies of risk factors and health outcomes.

## Background

Health researchers often use survey studies to examine associations between risk factors at one time point and health outcomes later in life. A possible threat to generalizability of findings from such studies is selective non-response [1]. When non-responders do not differ from responders, the situation is termed missing completely at random (MCAR) [2]. This situation does not lead to bias, only to decreased power due to reduced sample size. If responders and non-responders differ regarding variables with no missing values, the situation is termed missing at random (MAR) [2]. An example of this is when responders and non-responders differ in terms of age and gender, and this information is available to the researcher for non-responders as well as responders through a national registry. If non-response is related to variables with missing information, the situation is called missing not at random (MNAR) [2]. An example of this is when participants in a survey study about risk factors for poor health have better health or fewer risk factors than those who do not participate. MNAR is considered the most serious situation [2, 3].

Unlike MAR, treatment of MNAR requires accounting for the missing data mechanism [4]. Two of the most common approaches are selection models and pattern mixture models [4, 5]. In selection models, missingness is conditioned on study variables, and the joint distribution of missingness and the outcome is modeled. The Heckman sample selection model is a well-known example of this [6]. In pattern mixture models, parameters are assumed to differ between those who respond and those who do not respond, and the true population parameters are estimated as a mix of these [4].

Because the data do not provide information about the true missing data mechanism, it is advised to perform several analyses under different scenarios when treating MNAR [4, 7]. Multiple analyses could give information about the degree to which results differ depending on the missing data model, and the fit of different models can be compared. However, different fit criteria may suggest different models as best fitting [8], and different selection models may lead to similar fit, but to different results [4, 9]. Selection models and pattern mixture models can be highly sensitive to strong assumptions that are not readily testable [6]. Correct specification of the missingness model may be very important, but also very difficult [4].

Several detailed approaches for treating MNAR have been developed. For example, Ibrahim, Lipsitz and colleagues have proposed methods for including the missing data mechanisms into the log-likelihood of the study variables [7, 9–11]. It has been shown that valid results may be obtained when missing data are properly accounted for in this way [7, 11]. MNAR approaches have been implemented in several R packages. One example is the ‘brlrmr’ package for treating MNAR in the outcome of logistic regression models [12]. Another R package, miceMNAR, [13, 14], allows combining selection modeling with multiple imputation (MI) for treating MNAR in continuous and binary outcomes. Other important contributions in recent years are simulation studies performed for better understanding and improvement of criteria for selecting the true missingness model under MNAR [8], and comparison of different imputation methods for different missing mechanisms [15].

Despite available approaches for treating missing data under MNAR, most researchers seem not to take advantage of these methods. In fact, most medical researchers do not even apply methods for handling MAR, even if these methods are implemented in statistical software used by many researchers, such as SPSS and Stata. According to Ibrahim, Chu and Chen, the most common way of “dealing” with missing data in medical studies, is to analyze complete cases [16]. Little and colleagues reviewed 80 empirical studies published in the Journal of Pediatric Psychology in the year 2012. None mentioned treating MNAR, and only 13 studies used MAR methods such as MI and full information maximum likelihood (FIML). Almost half of the 80 studies did not mention missing data explicitly at all, and most of those who did, analyzed only complete cases [17]. Lang & Little found similar results when they reviewed 169 empirical studies published in Prevention Science between 2013 and 2015 [6]. Again, the most common method was complete case analysis. Ibrahim and colleagues argue that the reason for the common use of complete case analysis may be that this is the default of many statistical software packages [16]. Approaches for handling MNAR are not as easily available in standard software as are MAR methods (except for the Heckman methods). MNAR methods seem to require more statistical understanding, and researchers should make sure they have sufficient statistical expertise if they use them [4–6]. This may be particularly relevant regrading categorical variables. Little and Rubin warn that MNAR models for categorical variables may lead to more biased results than MAR models in some situations, even when data are MNAR [4]. Others warn that approaches appropriate in some situations (e.g. for continuous outcomes or for normally distributed variables), have been misused in situations where they are not valid (e.g. with binary outcomes or highly skewed variables) [4, 13, 18]. Such misuse of MNAR methods may introduce, rather than alleviate, bias [4].

The fact that most medical researchers do not treat missing data appropriately, and particularly not MNAR data, emphasizes the importance of preventing MNAR bias in the first place. Hence, the aim of the current paper is to guide researchers in how to prevent bias, in order to avoid creating MNAR situations that are not treated correctly.

Different MNAR situations may produce varying degrees of bias, from practically unbiased to very misleading estimates of associations [1, 13, 19–22]. We therefore need more knowledge about when MNAR leads to serious versus less serious bias. Previous studies have demonstrated that degree of dependency between non-response and study variables is directly linked to degree of bias in logistic regression with dichotomous outcomes as well as in linear regression with continuous outcomes [1, 13, 19, 22]. Further, missingness related to the outcome has been found to be particularly problematic in some studies [19, 23].

A further important distinction between different MNAR situations may be whether or not extreme cases are present in the sample. However, we lack knowledge about this. Previous studies have generally examined effects of underrepresentation of extreme cases (e.g. those who have heaviest health problems are under-represented). Findings about bias from studies where extreme cases are under-represented may not be generalized to studies where they are not present at all. Knowledge on the degree to which total absence of extreme cases leads to more biased estimates than under-representation of these cases may help researchers spend their resources in better ways, and may contribute to prevent bias in health studies.

Persons with low risk and low levels of health problems/diseases typically respond more often to survey studies than do persons with high risk of such problems/diseases [1, 24–29]. Hence, recruiting and retaining the latter participants may require more resources than recruiting and retaining the former. With the same amount of resources, researchers will thus probably get a higher response rate in a study by spending all their resources on recruiting as many persons as possible without doing any particular effort to recruiting and retaining persons with the highest levels of health problems. Even if researchers spend extra resources on recruiting and retaining some persons with extreme levels of health problems, these persons may still be under-represented in the sample. Hence, researchers may be left with a sample that is not fully representative and with lower response rate than if they had spent the resources on obtaining and retaining as many participants as possible from the group of people who typically respond to survey studies (i.e. persons who have lower levels of health problems). It is important to know the degree to which succeeding in recruiting and retaining at least some persons with extreme levels of health problems may prevent bias substantially or whether results are less biased only if high response rates are obtained.

Health survey studies often use ordinal scales to measure health outcomes (e.g. a five- or four-point scale ranging from ‘no problems’ to ‘very heavy problems’) [30–34]. Selective non-response may lead to skewed distributions and floor/ceiling effects in such variables, requiring analyzing them as ordered categorical variables [34, 35]. Previous studies have shown that missingness may be more difficult to handle in categorical than in continuous data [36, 37]. Hence, there seems to be a particular need for more knowledge about prevention of MNAR situations in studies using ordered categorical variables.

Data simulation studies are ideal for systematically investigating under what circumstances bias occurs [35]. This is because the researcher knows the true population values and can compare estimates obtained under different scenarios to these true population values [35].

*Aims of the study:* The main aim of the current study was to increase current knowledge on how to prevent biased estimates in longitudinal survey studies of associations between risk factors and health outcomes. More specifically, the aims were to examine relative bias in situations where 1) extreme scores are under-represented, but present in the sample and 2) extreme scores are totally missing, and to compare these situations. The difference between relative bias in these two types of situations suggests the potential for preventing bias by succeeding to include some individuals with the highest levels of health problems, compared to failing to include them. The aim was to examine this under different response rates, and different degrees of dependency between missingness and study variables.

The health outcome was simulated as a continuous trait in the population, measured with an ordered categorical scale by the researcher. This is in accordance with what is done in many survey studies [38–46] and in accordance with the assumption that the underlying liability of many complex diseases seems to be continuous [47]. Three predictors were modeled – two predicting the health outcome as well as missingness, and one additional predictor of missingness.

## Methods

Analyses were performed in Mplus version 8 [35] and in R version 3.4.3 [48]. First, populations were defined, and 500 random samples were drawn from each population. Analyses were performed on these 500 samples. The high number of samples was chosen to avoid random variation between samples to affect the results.

The four study variables (× 1, × 2, × 3, and the health outcome) were modeled as continuous and normally distributed traits with mean = 0 and variance 1.15. This variance was chosen to allow the residual variance of the health outcome to be 1. The associations between health outcome and predictors will be termed b_pred._ This association was set to be b_pred_ = 0.20 for × 1, b_pred_ = 0.30 for × 2, and b_pred_ = 0.00 for × 3 in the population. Predicted health outcome was thus given by:

y = 0.2*× 1 + 0.3*× 2 + random component

The random component was normally distributed with a mean of zero and variance fixed to 1. The choice of magnitude of the associations between predictors and health outcome ensured that studies with a sample size of 100–150 participants would have sufficient power to detect it. This makes the results relevant for many studies, not only those with very large samples. The size of the associations also approximates real life effects between social skills and later depressive symptoms as identified by Nilsen and colleagues [49].

Missingness was modeled in the health outcome, while predictors (× 1, × 2, and × 3) were completely observed. MNAR situations where extreme scores on the health outcome were under-represented, but present in the samples, were modeled in accordance with previous simulation studies of non-response [1, 22]. Different degrees of dependency between non-response and predictors and health outcome were modeled by constructing liability of non-response (termed L) as a latent normally distributed continuous variable with mean = 0 and variance = 1.15. Dependency between L and the study variables (× 1, × 2, × 3, and health outcome) is termed b_non._ This was set to vary from b_non_ = 0.10 for predictors (× 1, × 2, and × 3) and health outcome to a maximum of b_non_ = 0.30, in accordance with previous simulation studies [1, 22]. This dependency was given by:

L = b0 + b_non_1*× 1 + b_non_1*× 2 + b_non_1*× 3 + b_non_2*y + random normal component

The random component had a mean of zero and a variance of 1.15 minus the variance explained by × 1, × 2, × 3, and y. The random component thus had slightly different variance in the different conditions. This was done to ensure that all variables had the same total variance of 1.15 in all conditions, which makes regression coefficients more easily interpretable. In each situation, the association with liability of non-response was the same for all three predictors (× 1, × 2, and × 3), as indicated by the same regression coefficient (b_non_1) for all of them. A dichotomous missingness indicator was termed M, with the value 0 for individuals with data on the health outcome, and 1 for individuals without data on the outcome. In the 70% response-rate situation, M was 1 if the predicted value of the latent continuous liability of non-response (L) was > 0.52 standard deviations (SD) above the mean. In the 50% response-rate situation, M was 1 when predicted L was above the mean.

**Fig. 1:**
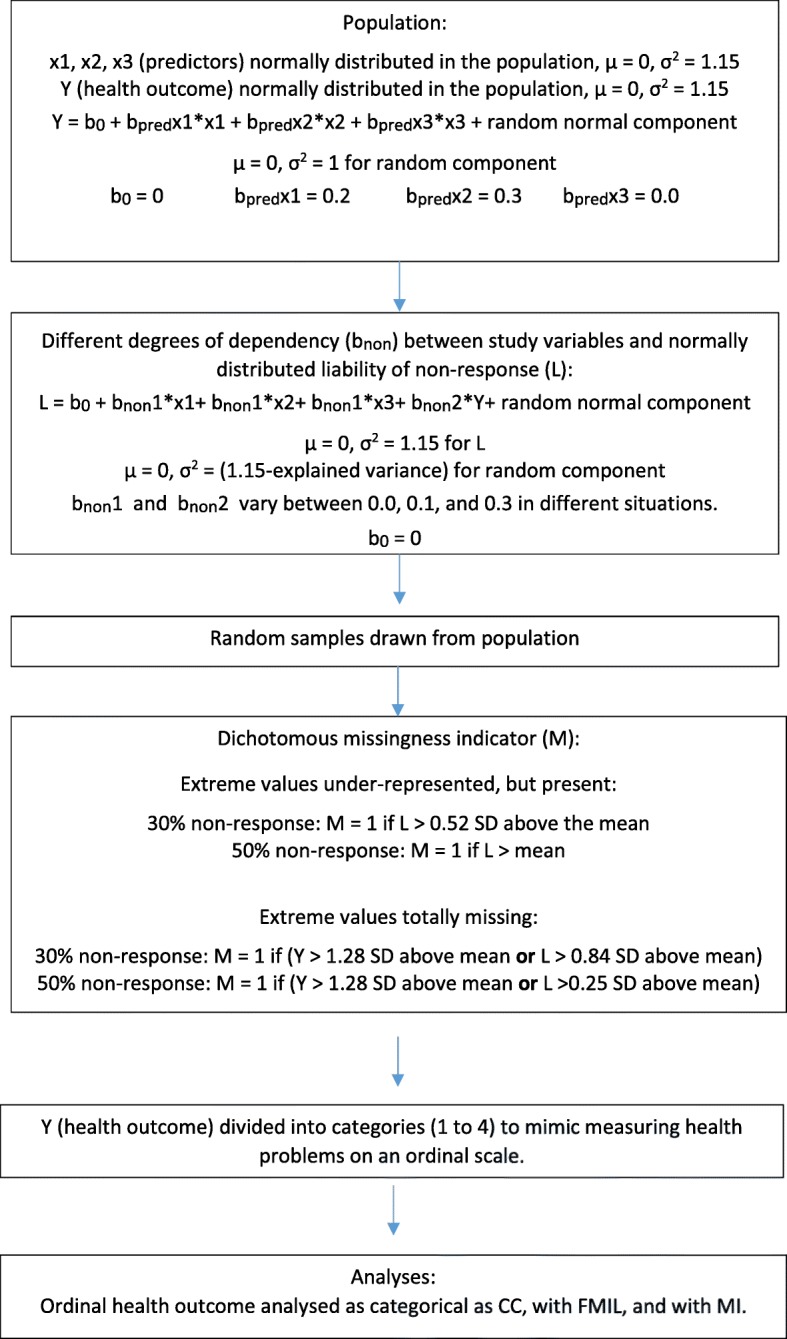
Overview of modeling the data. Notes: μ is population mean. σ^2^ is population variance. CC = complete case analysis, FIML = full information maximum likelihood, MI = multiple imputation

The 50% response rate mimics the mean response rate for large survey studies (*N* > 1000) reported in a review [50]. See Fig. 1 for an overview of modeling the data.

In the situations described above, the most extreme values on the health outcome (i.e. 10% highest values on the continuous health outcome) were underrepresented in the samples because missingness was related to the study variables. However, the extreme cases were not totally excluded.

To examine MNAR situations where none of the observations with the most extreme scores on the health outcome were present, the 10% highest values on the health outcome were removed. Additional non-response was modeled in the same way as above. Hence, in the 70% response rate situation, observations with the 10% highest scores on the health outcome were removed, and 20% of the observations were removed as in the situations above. M was thus 1 if predicted value on the health outcome was > 1.28 SD above the mean, or predicted value on L was > 0.84 SD above the mean. Varying degrees of dependency between study variables and L in the different scenarios lead to different degrees of overlap between the two criteria for missingness (predicted value on the outcome and predicted value on L). The cut-off value of L was changed accordingly, so that 30% of the observations were assigned M = 1.

The situation with 50% response rate was produced in the same way. M was 1 if predicted health outcome was > 1.28 SD above the mean or predicted L was > 0.25 SD above the mean. Again, different degrees of dependency between study variables and L made it necessary to vary the cut-off value for L, to ensure 50% non-response in all situations.

We wanted to mimic using an ordinal scale for measuring a trait that is normally distributed in the population. Hence, the health outcome variable that was defined as continuous and normally distributed in the population was used as basis for creating an observed ordinal health outcome variable. This ordinal observed health outcome variable was constructed with four categories. The first category corresponded to scoring one SD or more below the mean on the continuous normally distributed health outcome variable. The second category corresponded to scoring between one SD below the mean and the mean on that variable. The third category corresponded to scoring between the mean and one SD above the mean, and the fourth category corresponded to scoring one SD or more above the mean on the continuous normally distributed outcome variable. Hence, observations with values one SD or more below the mean on the continuous population health outcome variable, were allocated to the first category on the observed ordinal health outcome variable. Those with values between one SD below the mean and the mean on the continuous population outcome variable were allocated to category two on the observed ordinal outcome variable and so forth.

Analyses were performed with this ordinal observed health outcome treated as a categorical variable, using probit regression. The risk factor variables (× 1 and × 2) were predictors. Data were analyzed with complete cases (CC), with FIML and MI with 50 imputed data sets. CC and FIML analyses were performed in Mplus. × 3 was used as an auxiliary variable in FIML, and as predictor in the imputation model. Performing multiple imputations on each of 500 randomly drawn data sets required creating loops. MI and analysis of the MI data were therefore performed in R, using the mice package and the ‘pmm’ method (predictive mean matching) [51].

The probit regression model in Mplus assumes a normally distributed continuous latent response variable with residual variance fixed to one underlying the observed categorical variable [35]. This analytic model thus matched the simulated scenario where an ordinal variable was used for measuring a trait that was normally distributed in the population. Probit regression in Mplus provides standardized estimates that correspond to the association between a predictor and the normally distributed continuous underlying latent response variable (with mean = 0, residual variance = 1). The unbiased estimate from the probit regression was therefore the same as the population value of the association between the predictor and the normally distributed continuous population health outcome variable (i.e. b_pred_ × 1 = 0.20 and b_pred_ × 2 = 0.30). The association between the observed score on the ordinal variable and the estimated score on the underlying continuous normally distributed latent variable in probit regression is given in the following way: The probability (P) of obtaining a category (k) on the observed ordinal variable is:

$$ \mathrm{P}\ \left(\mathrm{ordinal}\ \mathrm{category}\ \mathrm{k}\ |\ \upmu, \upsigma, {\uptheta}_1,\dots \dots, {\uptheta}_{\mathrm{k}-1}\right)=\upphi\ \left(\uptheta \mathrm{k}\right)-\upphi\ \left(\uptheta \mathrm{k}-1\right) $$

where ϕ is the standardized cumulative normal function, μ is the mean and σ the standard deviation of the latent normally distributed underlying variable, and θ is the threshold [34]. For the first observed category, the threshold θk-1 on the latent normal underlying variable is negative infinity. For the highest observed category (k), the threshold θk is positive infinity [34].

To enable comparison of results from MI analyses performed in R to the CC and FIML results from Mplus, results from probit regression in R were standardized with respect to the latent underlying continuous health variable, in the same way as is done in Mplus:

$$ \mathrm{bs}=\mathrm{b}\ast \mathrm{SD}\left(\mathrm{x}\right)/\mathrm{SD}\left(\mathrm{u}\ast \right) $$

and

$$ \mathrm{SD}\left(\mathrm{u}\ast \right)=\mathrm{SQRT}\left({\mathrm{b}}^2\ast \mathrm{V}\left(\mathrm{x}\right)+1\right) $$

where bs is the standardized estimate, b is the unstandardized estimate, SD(x) is standard deviation of x, SD(u*) is standard deviation of the latent underlying continuous variable, and V(x) is variance of x [52].

Relative bias was calculated as the difference between the true population value (i.e. b_pred_ × 1 = 0.20 and b_pred_ × 2 = 0.30) and the estimate, divided by the true population value [35]. Difference between relative bias in the situation where extreme scores were totally missing versus situations where they were under-represented was calculated. Potential for preventing bias by ensuring that the 10% most extreme scorers are not totally missing from the sample, was calculated as this difference divided by the relative bias in the first situation and multiplied by 100:

$$ \mathrm{Potential}\ \mathrm{for}\ \mathrm{preventing}\ \mathrm{bias}=\frac{\mathrm{R}1-\mathrm{R}2\ }{\mathrm{R}1}\ast 100 $$

where R1 and R2 are relative bias in situations without and with extreme scores present, respectively.

The 95% coverage was also used to evaluate bias. This is a measure of the proportion of the randomly drawn samples that gives a 95% confidence interval containing the true population value. The higher the 95% coverage, the less risk of drawing a sample yielding a biased estimate.

The sample size before non-response was 1000 in all situations, the same as the baseline sample size in the TOPP study.

### Follow-up analyses

We then wanted to examine the degree to which results were specific to situations with four categories on the health outcome variable. The observed ordinal outcome variable was therefore modeled to have five categories and two categories. For five categories, four thresholds were defined (1 SD below the mean, 0.5 SD below the mean, 0.5 SD above the mean, and 1 SD above the mean on the population continuous normally distributed outcome variable). For two categories, one threshold was defined at the mean.

Analyses were also performed without using an ordinal variable for the health outcome. This was done to examine if results could be generalized from situations with an observed ordinal variable to situations with an observed continuous outcome variable. Linear regression analyses were then run with the continuous normally distributed health outcome variable, without constructing an ordinal variable. The follow-up analyses were performed for situations with 50% response rate.

## Results

**Fig. 2:**
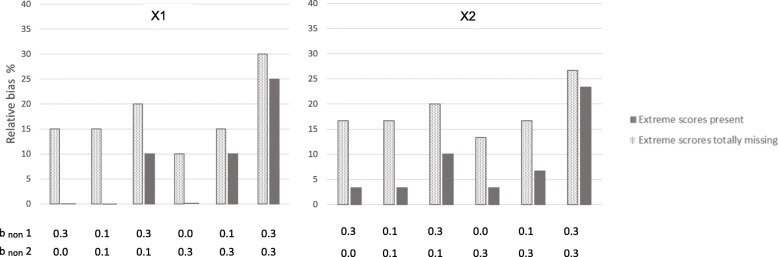
Relative bias in the associations between predictors and health outcome at 70% response-rate. Notes: × 1 to the left, and × 2 to the right. Solid bars represent situations where extreme scores are present even if they are under-represented in the samples. Dotted bars represent situations where the 10% most extreme scores on the health outcome are totally missing from the samples. b _non_ 1 = dependency between liability of non-response and each of the three predictors (× 1, × 2, × 3). b _non_ 2 = dependency between liability of non-response and the health outcome. Different degrees of dependency between study variables and liability of non-response are modeled. Relative bias is calculated by dividing the difference between the estimated and the true value on the true value of b_pred_ = 0.20 for × 1 and 0.30 for × 2

First, a probit regression model was run with the categorical observed outcome variable without any missing values. This showed that the unbiased results for b_pred_ × 1 was 0.20, and the unbiased b_pred_ × 2 was 0.30. These values were the same as those defined in the population for the normally distributed outcome variable of risk for health problems. The more the estimates with missing data deviate from these values, the more biased they are. The 95% coverage should be close to 95 for unbiased results. We will first comment on results from situations where extreme scores are totally missing from the sample. Next, results from situations where extreme scores are under-represented, but present will be commented. Comparison of these results indicates the potential for preventing bias by successfully including some individuals with the highest levels of health problems, compared to failing to include these persons. These two types of situations are presented next to each other in Tables 1 and 2, and in Figs. 2 through 5, for ease of comparing results from samples *with* versus *without* extreme scores present. We then present the proportion of bias in the situations without extreme cases present that can be prevented if the extreme cases are not totally missing from the sample.

**Table 1:** Estimates of associations between predictors and health outcome at 70% response-rate

b_non_			10% most extreme values on the health outcome totally missing						Extreme values on the health outcome not totally missing					
			CC		FIML		MI		CC		FIML		MI	
×1,×2,× 3	Y	Pred.	B_pred_ (SE)	95% coverage	B_pred_ (SE)	95% coverage	B_pred_ (SE)	95% coverage	B_pred_ (SE)	95% coverage	B_pred_ (SE)	95% coverage	B_pred_ (SE)	95% coverage
0.3	0.0	×1	.17 (.04)	88	.18 (.04)	92	.17 (.04)	86	.20 (.04)	92	.20 (.04)	92	.20 (.04)	95
		×2	.25 (.04)	86	.26 (.04)	92	.25 (.04)	76	.29 (.04)	94	.30 (.04)	93	.29 (.04)	95
0.1	0.1	×1	.17 (.04)	88	.17 (.04)	91	.17 (.04)	88	.20 (.04)	94	.20 (.04)	94	.20 (.04)	95
		×2	.25 (.04)	81	.26 (.04)	91	.25 (.04)	79	.29 (.04)	95	.29 (.04)	93	.29 (.04)	96
0.3	0.1	×1	.16 (.04)	84	.17 (.04)	88	.16 (.04)	83	.18 (.04)	93	.19 (.04)	93	.18 (.04)	93
		×2	.24 (.04)	80	.25 (.04)	88	.24 (.04)	69	.27 (.04)	93	.28 (.04)	95	.28 (.04)	91
0.0	0.3	× 1	.18 (.04)	88	.18 (.04)	91	.17 (.04)	88	.20 (.04)	95	.20 (.04)	93	.20 (.04)	96
		×2	.26 (.04)	84	.26 (.04)	93	.26 (.04)	82	.29 (.04)	97	.29 (.04)	94	.29 (.04)	98
0.1	0.3	×1	.17 (.04)	88	.17 (.04)	88	.17 (.04)	88	.18 (.04)	93	.19 (.04)	95	.18 (.04)	94
		×2	.25 (.04)	77	.25 (.04)	88	.25 (.04)	76	.28 (.04)	93	.28 (.04)	93	.28 (.04)	93
0.3	0.3	×1	.14 (.04)	70	.14 (.04)	72	.13 (.04)	63	.15 (.04)	75	.15 (.04)	78	.14 (.04)	71
		×2	.22 (.04)	56	.23 (.04)	71	.21 (.04)	48	.23 (.04)	74	.24 (.04)	83	.23 (.04)	59

The true population values are b_pred_ ×1 = 0.20, b_pred_ ×2 = 0.30. Different degrees of dependency between study variables and non-response are modeled.

Y = health outcome. b_non_ = regression coefficients of normally distributed liability of non-response (L) on predictors (× 1, × 2, ×3) and on health outcome.

b_pred_ = coefficients for the regression of health outcome on × 1 and × 2, when the outcome is treated as a categorical variable, and the probit-link is used.

*SE* standard error, *95% coverage* percentage of the randomly drawn samples providing a 95% confidence interval containing the true population value. *FIML* full information maximum likelihood, *MI* multiple imputation (predictive mean matching). N in the original sample before non-response = 1000. × 3 was included as auxiliary variable in FIML and as predictor in MI. 50 data sets were imputed.

**Table 2:** Estimates of associations between predictors and health outcome at 50% response-rate

b_non_			10% most extreme values on the health outcome totally missing						Extreme values on the health outcome not totally missing					
			CC		FIML		MI		CC		FIML		MI	
×1,×2,× 3	Y	Pred.	B_pred_ (SE)	95% coverage	B_pred_ (SE)	95% coverage	B_pred_ (SE)	95% coverage	B_pred_ (SE)	95% coverage	B_pred_ (SE)	95% coverage	B_pred_ (SE)	95% coverage
0.3	0.0	×1	.18 (.05)	91	.18 (.05)	92	.17 (.05)	88	.20 (.04)	92	.20 (.05)	92	.20 (.05)	93
		×2	.25 (.05)	91	.26 (.05)	93	.25 (.05)	76	.29 (.04)	94	.30 (.04)	94	.29 (.05)	93
0.1	0.1	×1	.17 (.05)	89	.17 (.05)	91	.17 (.05)	88	.20 (.04)	92	.20 (.04)	92	.19 (.05)	93
		×2	.25 (.04)	89	.26 (.05)	93	.25 (.05)	82	.29 (.04)	96	.29 (.04)	95	.29 (.05)	95
0.3	0.1	×1	.16 (.05)	88	.17 (.05)	90	.16 (.05)	84	.18 (.05)	92	.18 (.05)	92	.18 (.05)	90
		×2	.24 (.05)	84	.25 (.05)	89	.23 (.05)	69	.27 (.04)	92	.28 (.05)	95	.27 (.05)	86
0.0	0.3	×1	.18 (.05)	90	.18 (.05)	93	.17 (.05)	91	.20 (.04)	94	.20 (.04)	94	.19 (.05)	95
		×2	.26 (.04)	90	.26 (.05)	94	.26 (.05)	85	.29 (.04)	95	.29 (.04)	93	.29 (.05)	95
0.1	0.3	×1	.16 (.05)	88	.17 (.05)	91	.16 (.05)	86	.18 (.05)	92	.18 (.05)	93	.18 (.05)	92
		×2	.24 (.04)	82	.25 (.05)	91	.24 (.05)	78	.27 (.04)	93	.28 (.04)	95	.27 (.05)	92
0.3	0.3	×1	.12 (.05)	68	.12 (.05)	69	.11 (.05)	54	.13 (.05)	70	.13 (.05)	72	.12 (.05)	60
		×2	.20 (.05)	58	.21 (.05)	68	.19 (.05)	43	.21 (.05)	68	.22 (.05)	76	.20 (.05)	49

The true population values are b_pred_ ×1 = 0.20, b_pred_ ×2 = 0.30. Different degrees of dependency between study variables and non-response are modeled

Y = health outcome. b_non_ = regression coefficients of normally distributed liability of non-response (L) on predictors (× 1, × 2, x3) and on health outcome

b_pred_ coefficients for the regression of health outcome on × 1 and × 2, when the outcome is treated as a categorical variable, and the probit-link is used

*SE* standard error, *95% coverage* percentage of the randomly drawn samples providing a 95% confidence interval containing the true population value. *FIML* full information maximum likelihood, *MI* multiple imputation (predictive mean matching). N in the original sample before non-response = 1000. X3 was included as auxiliary variable in FIML and as predictor in MI. 50 data sets were imputed

### Extreme scores of the health outcome totally missing

**Fig. 3:**
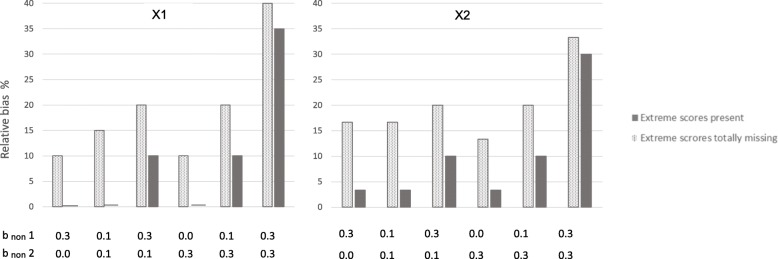
Relative bias in the associations between predictors and health outcome at 50% response-rate. Notes: × 1 to the left, and × 2 to the right. Solid bars represent situations where extreme scores are present even if they are under-represented in the samples. Dotted bars represent situations where the 10% most extreme scores on the health outcome are totally missing from the samples. b_non_ 1 = dependency between liability of non-response and each of the three predictors (× 1, × 2, × 3). b _non_ 2 = dependency between liability of non-response and the health outcome. Different degrees of dependency between study variables and liability of non-response are modeled. Relative bias is calculated by dividing the difference between the estimated and the true value on the true value of b_pred_ = 0.20 for × 1 and 0.30 for × 2

We modeled situations where the 10% most extreme scores on the health outcome were totally missing from the samples. Hence, these were situations in which the liability of non-response (L) was dependent on the predictors and outcome with persons with low scores being more likely to respond than persons with higher scores, and in addition none of those with the 10% highest scores on the health outcome had responded at all. The dotted bars in Figs. 2 and 3 show that estimates are clearly biased in all situations where the 10% most extreme cases are removed. This is true at 70 and 50% response rates. Figure 2 shows results for × 1 and × 2 at 70% response rate, Fig. 3 for × 1 and × 2 at 50% response rate. The figures show that even if non-response is only weakly related to the study variables (e.g. dependency between L and predictors and outcome is b_non_ = .10) for the majority of the missingness, results are clearly biased when the 10% most extreme scores are missing. All estimates were biased between 10 and 40%. Relative bias increased as dependency between L and study variables increased, and was highest when b_non_ was 0.30 for both health outcome and predictors. More details are given in Tables 1 and 2 (showing 70 and 50% response rate, respectively). Tables 1 and 2 also show that results were similar for complete case analysis, FIML and MI.

**Fig. 4:**
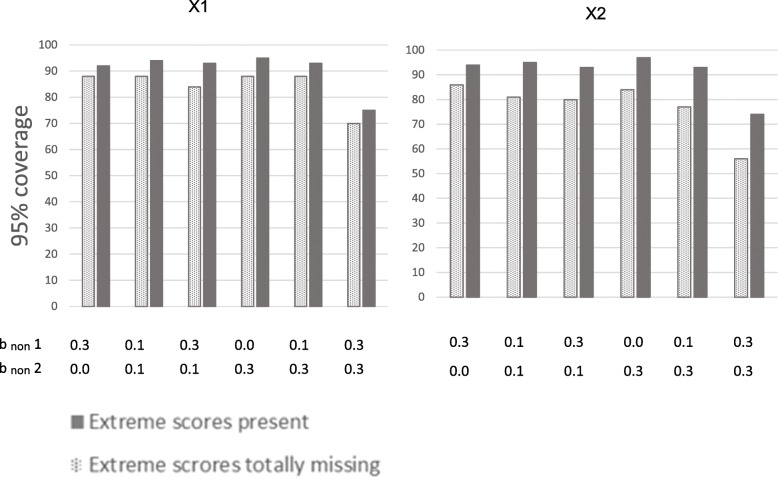
95% coverage of the associations between predictors and health outcome at 70% response-rate. Notes: Illustration of 95% coverage of the estimates of the associations between predictors (× 1 and × 2) and health outcome in situations with different degrees of dependency between study variables and liability of non-response. × 1 to the left, and × 2 to the right. Solid bars represent situations where extreme scores are present even if they are under-represented in the samples. Dotted bars represent situations where the 10% most extreme scores on the health outcome are totally missing from the samples. 95% coverage is the proportion of samples randomly drawn from the population that has a 95% confidence interval containing the true population value of b_pred_ × 1 = 0.20, and b_pred_ × 2 = 0.30. b_non_ 1 is dependency of liability of non-response on predictors (× 1, × 2, and × 3).b_non_ 2 is dependency of liability of non-response on health outcome

**Fig. 5:**
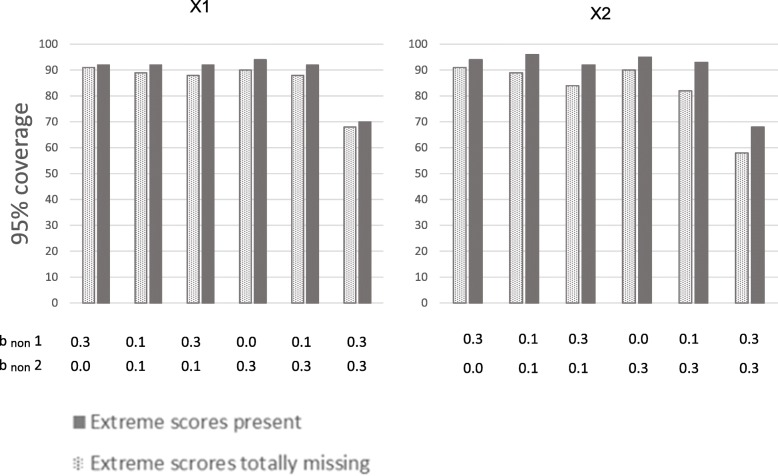
95% coverage of the associations between predictors and health outcome at 50% response-rate. Notes: Illustration of 95% coverage of the estimates of the associations between predictors (× 1 and × 2) and health outcome in situations with different degrees of dependency between study variables and liability of non-response. × 1 to the left, and × 2 to the right. Solid bars represent situations where extreme scores are present even if they are under-represented in the samples. Dotted bars represent situations where the 10% most extreme scores on the health outcome are totally missing from the samples. 95% coverage is the proportion of samples randomly drawn from the population that has a 95% confidence interval containing the true population value of b_pred_ × 1 = 0.20, and b_pred_ × 2 = 0.30.b_non_ 1 is dependency of liability of non-response on predictors (× 1, × 2, and × 3).b_non_ 2 is dependency of liability of non-response on health outcome

Figures 4 and 5 show the 95% coverage of the estimates (dotted bars). Figure 4 shows coverage for × 1 and × 2 at 70% response rate, and Fig. 5 at 50% response rate. All 95% coverages were below 90% for × 1 and × 2 when response rate was 70%. When response rate was 50%, the highest 95% coverages were 91% for × 1 and × 2, and most 95% coverages were below 90%.

### Extreme scores on health outcome under-represented, but not totally missing

The solid bars in Figs. 2 and 3 show results for situations where extreme scores are under-represented, but not totally missing. Figure 2 shows results at 70% response rate, and Fig. 3 at 50% response rate. The solid bars in the figures show that estimates are unbiased or weakly biased in several of the situations. Estimates for × 1 were unbiased when liability of non-response (L) was relatively weakly related to study variables (b_non_1 = 0.1 and b_non_2 = 0.1), and when L was related to predictors (× 1, × 2, × 3) *or* to the health outcome. Estimates for × 2 were weakly biased (< 4%) in these situations. This was true for 70 and 50% response rates. Bias increased as dependency between L and study variables got stronger. When dependency was b_non_ = .30 between L and both health outcome and predictors, estimates of associations between risk factor and health outcome were clearly biased at both response rates (70 and 50%, but more biased with lower response rate). Estimates are relatively similar across the 70 and 50% response rates when extreme scores are present in the sample (solid bars). Tables 1 and 2 show more details. These tables also show that results from complete case analysis, FIML and MI are similar. Table 1 shows results at 70% response rate and Table 2 at 50% response rate.

Figures 4 and 5 show 95% coverage for the estimates (solid bars), for × 1 and × 2 at 70 and 50% response rates, respectively. 95% coverages exceeded 90% for × 1 and × 2 in all situations, except when b_non_ = .30 for both health outcome and predictors. This was true for 70 and 50% response rates.

### Potential for preventing bias

**Fig. 6:**
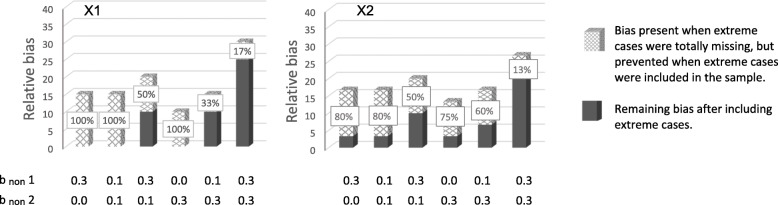
Bias prevented when extreme cases were not totally missing (70% response-rate). Notes: × 1 to the left, and × 2 to the right. The x-patterned parts of the bars show relative bias that was present when extreme cases were totally missing from the sample, but that was not present in the situations where extreme values were under-represented but not totally missing. In other words, this is relative bias that was prevented by having persons with extreme values in the sample, even if they were under-represented. Dark grey parts of the bars show bias still present when extreme cases were in the sample. When there is no dark grey part of a bar, all of the bias (100%) was prevented by not totally excluding extreme scores. The sum of the x-patterned and dark grey parts of a bar, is the total amount of relative bias present in situations where extreme scores were totally missing from the sample. The percentages in the figure were calculated by dividing the difference in relative bias in the two situations by the amount of relative bias in the situations without extreme cases, and multiplied by 100 Percentage prevented bias $=\frac{\mathbf{R1}-\mathbf{R}\mathbf{2}\ }{\mathbf{R1}}\ast \mathbf{100}$ where R1 and R2 are relative bias in situations without and with extreme scores present, respectively. b_non_ 1 is dependency of liability of non-response on predictors (× 1, × 2, and × 3).b_non_ 2 is dependency of liability of non-response on health outcome

**Fig. 7:**
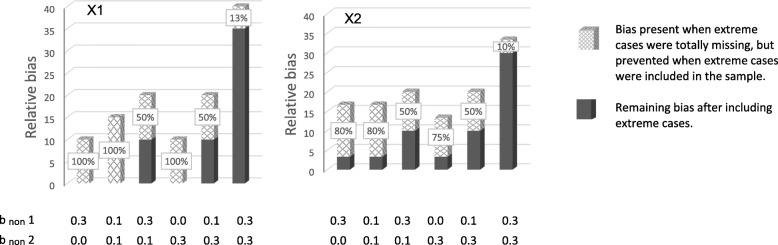
Bias prevented when extreme cases were not totally missing (50% response-rate). Notes: × 1 to the left, and × 2 to the right. The x-patterned parts of the bars show relative bias that was present when extreme cases were totally missing from the sample, but that was not present in the situations where extreme values were under-represented but not totally missing. In other words, this is relative bias that was prevented by having persons with extreme values in the sample, even if they were under-represented. Dark grey parts of the bars show bias still present when extreme cases were in the sample. When there is no dark grey part of a bar, all of the bias (100%) was prevented by not totally excluding extreme scores. The sum of the x-patterned and dark grey parts of a bar, is the total amount of relative bias present in situations where extreme scores were totally missing from the sample. The percentages in the figure were calculated by dividing the difference in relative bias in the two situations by the amount of relative bias in the situations without extreme cases, and multiplied by 100 Percentage prevented bias $=\frac{\mathbf{R1}-\mathbf{R}\mathbf{2}\ }{\mathbf{R1}}\ast \mathbf{100}$ where R1 and R2 are relative bias in situations without and with extreme scores present, respectively. b_non_ 1 is dependency of liability of non-response on predictors (× 1, × 2, and × 3). b_non_ 2 is dependency of liability of non-response on health outcome

Potential for preventing bias by not totally excluding individuals with extreme values from the sample is shown in Figs. 6 and 7. Figure 6 shows prevention of relative bias at 70% response rate, and Fig. 7 at 50% response rate. The x-patterned parts of the bars show relative bias that was present when extreme cases were totally missing from the sample, but that was not present in the situations where extreme values were under-represented but not totally missing. In other words, this is relative bias that was prevented by having persons with extreme values in the sample, even if they were under-represented. The dark grey parts of the bars show relative bias that was present even when extreme cases were in the sample. When there is no dark grey part of a bar, all of the bias (100%) was prevented by not totally excluding extreme scorers. When the dark grey part and the x-patterned part are of equal size, 50% of the bias was prevented. The sum of the dark grey part and the x-patterned part, is the total amount of relative bias when extreme scores were totally missing from the sample.

The figures show that the potential for preventing bias was 100% in several situations, and 50–80% in other situations. Hence, in some situations, there was no bias left when extreme scorers were in the sample (100% of bias prevented), even if they were under-represented. In other situations 20% or 50% of the bias was left when extreme cases were in the sample (80% or 50% of the bias was prevented, respectively). When dependency between liability of non-response and study variables was relatively strong for both health outcome and predictors, the potential for preventing bias was lower, but never less than 10%.

### Follow-up analyses

**Fig. 8:**
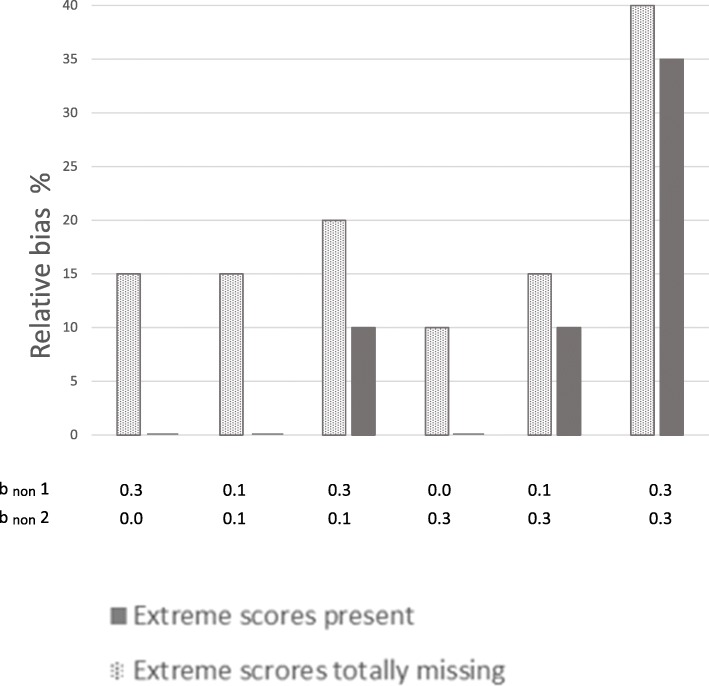
Relative bias in the association between × 1 and health outcome with 5 categories.Notes: Results are from situations with 50% response-rate. Solid bars represent situations where extreme scores are present even if they are under-represented in the samples. Dotted bars represent situations where the 10% most extreme scores on the health outcome are totally missing from the samples. Different degrees of dependency between study variables (× 1, × 2, × 3, and health outcome) and liability of non-response were modeled. b_non_ 1 is dependency of liability of non-response on predictors (× 1, × 2, and × 3). b_non_ 2 is dependency of liability of non-response on health outcome

**Fig. 9:**
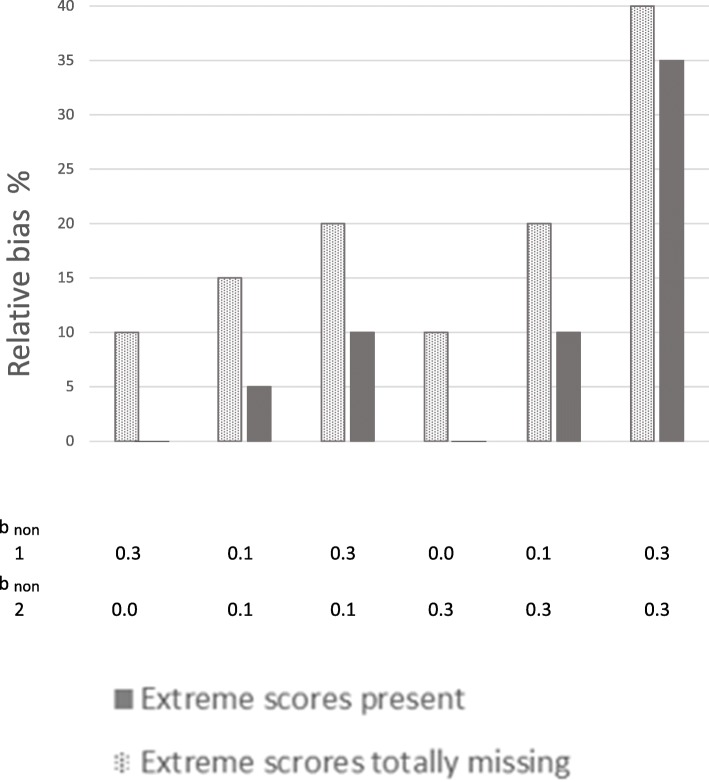
Relative bias in the association between × 1 and health outcome with 2 categories. Notes: Results are from situations with 50% response-rate. Solid bars represent situations where extreme scores are present even if they are under-represented in the samples. Dotted bars represent situations where the 10% most extreme scores on the health outcome are totally missing from the samples. Different degrees of dependency between studyvariables (× 1, × 2, × 3, and health outcome) and liability of non-response were modeled. b_non_ 1 is dependency of liability of non-response on predictors (× 1, × 2, and × 3). b_non_ 2 is dependency of liability of non-response on health outcome

Follow-up analyses were performed to examine if the number of categories on the observed ordinal outcome variable affected the results. Figures 8 and 9 show results from dividing the health outcome variable into five and two categories, respectively. Results for × 1 at 50% response rate are shown. The figures show that results were similar regardless of number of categories on the ordinal outcome variable.

**Fig. 10:**
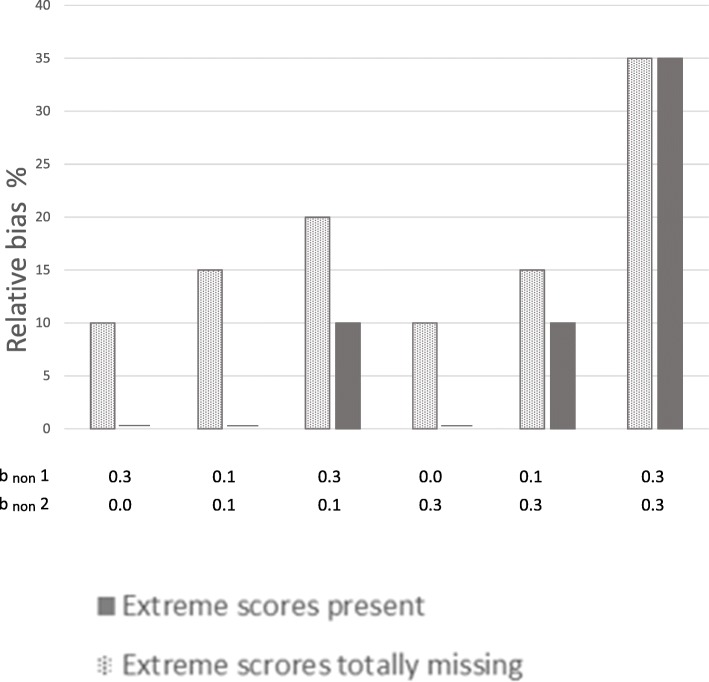
Relative bias in the association between × 1 and continuous health outcome. Notes: Results are from situations with 50% response-rate. Solid bars represent situations where extreme scores are present even if they are under-represented in the samples. Dotted bars represent situations where the 10% most extreme scores on the health outcome are totally missing from the samples. Different degrees of dependency between study variables (× 1, × 2, × 3, and health outcome) and liability of non-response were modeled. b_non_ 1 is dependency of liability of non-response on predictors (× 1, × 2, and × 3). b_non_ 2 is dependency of liability of non-response on health outcome

Follow-up analyses were also performed to examine if results were generalizable to situations where an observed continuous outcome variable was used. The results for × 1 at 50% response rate are shown in Fig. 10. The finding that results were clearly more biased when extreme scores were totally missing from than sample than when extreme scores were under-represented but present, also applied to this situation.

## Discussion

The main aim of the current study was to increase current understanding of how health researchers can prevent bias in estimates from longitudinal survey studies. Previous studies have shown that MNAR situations can lead to serious bias in association estimates. The current study added to this by showing that the degree of bias in MNAR situations may vary from mild to very serious, depending on whether at least some individuals with the heaviest health problems are included in the sample. These results suggest that bias due to MNAR to a large extent can be prevented if researchers manage to recruit and retain at least some individuals with extreme levels of health problems. Results from Monte Carlo simulations showed that estimates of longitudinal associations between a predictor and a health outcome were relatively robust against selective non-response when at least some extreme values of the health outcome were present in the sample, even if missingness was MNAR, extreme cases were under-represented, and response rate was relatively low. Estimates were clearly biased even at high response rates when extreme values were totally missing.

Several studies have shown that most medical researchers only analyze complete cases, despite the fact that approaches for treating MNAR are available [6, 16, 17]. This emphasizes a need for more information about how to prevent bias due to MNAR. Previous real life studies have shown mixed results regarding the degree to which association estimates are biased due to selective non-response, with some studies reporting serious bias and other studies reporting relatively unbiased results [29, 53, 54]. A recent study showed that missingness on the outcome variable may lead to serious bias [19]. The current findings may contribute to an increased understanding of how researchers can prevent bias, even if the sample is not perfectly representative of the population.

First, the current findings demonstrate that simply increasing response-rate, without regards to who the responders are, is not an effective way to prevent bias. The results showed that a study with 70% response rate can yield much more biased results than a study with only 50% response rate, even if data in both studies are MNAR.

Second, the findings showed that MNAR leads to increasing degrees of bias when the association between non-response and study variables gets stronger. This is in accordance with previous research on continuous and dichotomous variables [1, 13, 19, 22], and the current results showed that this was also the case for studies using ordinal categorical outcome variables. When the association between non-response and study variables was substantial (b_non_ = 0.3) for both predictors and health outcome, association estimates were clearly biased, even at a high (70%) response rate. When this association was weak for both predictors and health outcome, bias was mild or non-existent, even at a low (50%) response rate. When non-response was substantially related to outcome *or* predictors, but not to both predictor and outcome, bias was also relatively mild or non-existent.

Third, the current findings demonstrated that bias to a large extent can be prevented if persons with extreme levels of health problems are successfully recruited and retained in the samples. In some situations, 100% of the bias could be prevented by including some extreme values in the sample, and 50–80% could be prevented in other situations. The impact of MNAR depended heavily on whether or not at least some extreme values on the health outcome were included in the sample. When the 10% most extreme scores on the health outcome were totally missing, all estimates were biased, most of them between 10 and 20%, and some as much as 40%. Hence, an important distinction between different MNAR situations may be whether at least some of the most troubled individuals are present in the sample. When they are not, bias may be more severe than what is indicated by previous studies examining bias in situations where the most troubled individuals are under-represented, but present in the sample. This shows that recruiting and retaining at least some participants with extreme values on the health outcome may have dramatic positive effects on preventing bias in association estimates.

Follow-up analyses showed that including some individuals with extreme scores in the sample contributed to preventing bias in different situations (i.e. when using an ordinal outcome measure with different numbers of categories as well as when using a continuous outcome measure).

These results imply that researchers should spend more resources on recruiting and retaining persons with known risk of non-response (high scores on health problems) than on recruiting and retaining more of those persons typically responding (low scores on health problems). Journal editors considering papers should be more critical to studies where there is no examination of level of selectiveness of non-response than to studies with low response rates where there have been thorough examinations of the selectiveness of non-response. Further, the most important issue may not be whether the sample is fully representative of the population, but whether the range of values from the population is represented in the sample.

To get an idea of whether or not extreme scores on health outcomes are present in a population-based sample, health researchers could for example compare the highest levels of health problems in their sample to problem levels in clinical samples. If the population-based sample contains some values matching high values from clinical samples, the researchers might not need to worry too much about whether these high values are under-represented in the sample.

The two predictors used as risk factors for the health outcome as well as an additional third covariate predicting missingness, were included in the FIML and MI models. Applying FIML and MI did not change results noteworthy. This was expected as these were MAR methods, and missingness was MNAR. Also, the third predictor added to FIML and MI was only weakly associated with one of the two risk factors.

### Limitations

The current study only examined some selected scenarios that were believed to be relevant for real life research, and we cannot conclude that the findings will apply to all other types of situations. However, to increase generalizability to different situations, we varied several dimensions and examined effects of non-response under different levels of response rates and different levels of dependency between non-response and risk factors and health outcome. We also performed follow-up analyses where number of categories of the observed ordinal outcome variable varied, as well as analyses using an observed continuous outcome. Nevertheless, it is important to emphasize that the current results should not be generalized to situations that differ substantially from those examined here.

A further limitation is that the dependency between non-response and study variables is not known in real-life studies. Nevertheless, the current results emphasize the importance of using available information to examine and report the degree of selectiveness in non-response rather than only reporting non-response rates.

Statistical power has not been an issue in the current study, as statistical power as a function of sample size is easily calculated using standard software. Researchers must ensure that they have large enough samples to be able to detect associations they examine.

## Conclusions

The current results showed that MNAR data might lead to very different degrees of bias in estimates of associations between risk factors and health outcomes, depending on whether or not at least some of those with the heaviest health problems are included in the study. This knowledge might guide researchers in preventing biased estimates of longitudinal associations between risk factors and health outcomes. Association estimates may in many situations be valid even if data are MNAR and extreme scores on the health outcome are under-represented in the sample, as long as they are not totally missing. This implies that health researchers should spend resources on recruiting and retaining at least some participants with high scores on health problems rather than prioritizing recruiting very large numbers of those who typically respond to survey studies (i.e. persons with low scores on risk factors and health problems). This may contribute to preventing bias by 80–100% in many situations. The main question regarding generalizability may not be if the sample is totally representative of the population, or if data are MNAR or not, but whether or not at least some extreme values are present in the sample. Spending resources on increasing response rate, without making special efforts to include and retain the most troubled individuals, is not an effective way to prevent bias.

## Data Availability

Data can be made available by request to the first author.

## Abbreviations

b_non_: Dependency between non-response and study variables
b_pred_: Association between risk factor and health outcome
CC: Complete case analysis
FIML: Full information maximum likelihood
MAR: Missing at random
MCAR: Missing completely at random
MI: Multiple imputation
MNAR: Missing not at random
pmm: Predictive mean matching
SD: Standard deviation
SE: Standard error
